# Coagulation factor with potential for the treatment of heart failure

**DOI:** 10.1002/ctm2.1144

**Published:** 2022-12-13

**Authors:** Yang Cao, Aldons J. Lusis

**Affiliations:** ^1^ Department of Medicine/Division of Cardiology University of California Los Angeles California USA; ^2^ Human Genetics University of California Los Angeles California USA; ^3^ Microbiology, Immunology Molecular Genetics University of California Los Angeles California USA

1

We recently found that coagulation factor XI (FXI), a protein exclusively produced by the liver, plays an unexpected role in the heart.^1^ In the mouse model of heart failure with preserved ejection fraction (HFpEF), mice overexpressing FXI in the liver showed improved diastolic function and reduced inflammation and fibrosis. FXI overexpression activated the bone morphogenetic protein (BMP)‐SMAD1/5 pathway in the heart. The action of FXI on the heart requires proteolytic activity, as point mutations in its catalytic domain eliminated effects on BMP signalling and heart function. BMP7 is secreted as an inactive precursor that binds to the extracellular matrix, and our results indicated that it was cleaved by FXI, releasing the active growth factor from the pro‐domain. Results from human cohorts indicated that FXI has a similar function in humans. Our study identifies FXI as an endocrine factor that influences heart function, distinct from its role in coagulation. This previously unrecognized liver‐heart interaction enriches our understanding of liver‐heart communication and suggests a promising therapeutic strategy for heart failure.

Inter‐tissue communication via secreted proteins is a vital mechanism for proper physiologic homeostasis. We utilized a bioinformatics framework that uses natural variation in transcript levels across tissues in a panel of approximately 100 inbred strains of mice, termed the hybrid mouse diversity panel (HMDP), to identify and functionally annotate novel circuits of tissue‐tissue communication. In this study, global transcriptomic data from the liver and heart were generated across the HMDP mice and were used to detect the correlations between the endocrine factors from the liver and their downstream effects on the heart. Our analysis of expression data suggests that this method also applies to human populations. This approach was validated by in vitro and in vivo experiments and was supported by the fact that some of the predicted endocrine circuits are already known. Our datasets also provide pathway enrichment analysis to define the potential molecular targets of the endocrine factors. Collectively, the cross‐tissue predictions dissect molecular basis, including metabolic disorders and systemic inflammation, in complex human diseases.

FXI is a key component of the intrinsic pathway of blood coagulation. It acts downstream of FXII and triggers the middle phase of the intrinsic pathway of blood coagulation by activating FXI.^2^ FXI deficiency in patients generally does not cause spontaneous bleeding, as FXI is not required for the initial thrombin generation step.^3^ This is consistent with the possibility that it exerts other functions beyond blood coagulation. FXI has been found to protect against sepsis in part by enhancing the phagocytic capacity of neutrophils, a mechanism that is independent of activation via FXIIa.^4^ A number of studies revealed the relationship between FXI and stroke, coronary heart disease, and ischemic cardiomyopathy.^5^ In addition, the observation that mice with FXI overexpression exhibit other systemic metabolic benefits, including less body fat gain and improved lipid homeostasis, suggests potential effects of FXI beyond the heart. Whether other organs respond to FXI and whether these additional effects are mediated by the BMP7‐SMAD1/5 pathway need further investigation.

The fact that FXI is a direct mediator of liver‐heart cross‐talk suggests the possibility of therapeutic applications in heart failure. Recently, FXI has emerged as an important target for the development of anticoagulants with reduced bleeding risk, and a number of FXI or activated FXI inhibitors are now in clinical trials.^6^ For example, asundexian, a novel small molecule activated FXI inhibitor, might reduce thrombosis with a mild effect on haemostasis.^6^ Previously, FXI was not considered a serious therapeutic target for thrombosis, since its deficiency does not induce severe bleeding phenotype. Inhibiting FXI would be safe without the risk of severe bleeding, but thrombosis prevention would not be as effective as other anticoagulants (such as heparin). Recently, it has been suggested that FXI is more important for thrombosis than for hemostasis. These findings make FXI an ideal target to reduce venous thrombosis while minimizing the risk of severe bleeding. Therefore, the interest in FXI has increased dramatically. However, the protective role of FXI in HFpEF raises the possibility that inhibiting FXI might lead to untoward effects on heart function, systemic inflammation, and metabolism. Therefore, further mechanistic investigations and careful clinical evaluations are needed to fully understand the function of FXI.

Increased concentrations of plasma FXI are associated with an increased risk of thrombosis and ischemic stroke, suggesting that increasing FXI would be problematic as a therapeutic strategy.^7^ However, the downstream BMP‐SMAD1/5 pathway could serve as a potential therapeutic target. Prior studies have implicated BMP and SMAD pathways in traits relevant to heart failure. It has been reported that the BMP pathway was enriched for HFpEF but not HFrEF.^8^ Therapeutic strategies that enhance the myocardial BMP7‐SMAD pathway have yielded beneficial effects in mitigating the pathological remodelling in pressure overload and diabetic cardiomyopathy.^9^ In addition, BMP2 has been found to alleviate heart failure with type 2 diabetes by suppressing inflammasome signalling.^10^ BMP2 was inversely correlated with the levels of atrial natriuretic peptide and brain natriuretic peptide in patients with chronic heart failure. Taken together, the downstream BMP‐SMAD pathway exerts beneficial effects and may serve as potential therapeutic applications.

In summary, using our systems genetics approach with the HMDP cohort of mice combined with in vitro and in vivo studies, we found that liver‐derived FXI specifically activates the BMP‐SMAD1/5 pathway in cardiomyocytes, attenuating inflammation, fibrosis, and diastolic dysfunction in the context of an HFpEF model. These findings provide insights into clinical trials of therapeutic management targeting FXI and BMP‐SMAD pathways but suggest careful clinical evaluations.

## CONFLICT OF INTEREST

The authors declare that they have no conflict of interest.
